# Phenyl sulfate, indoxyl sulfate and *p*-cresyl sulfate decrease glutathione level to render cells vulnerable to oxidative stress in renal tubular cells

**DOI:** 10.1371/journal.pone.0193342

**Published:** 2018-02-23

**Authors:** Takeo Edamatsu, Ayako Fujieda, Yoshiharu Itoh

**Affiliations:** Pharmaceuticals & Agrochemicals Division, Kureha Corporation, Tokyo, Japan; University of PECS Medical School, HUNGARY

## Abstract

In chronic kidney disease patients, oxidative stress is generally associated with disease progression and pathogenesis of its comorbidities. Phenyl sulfate is a protein-bound uremic solute, which accumulates in chronic kidney disease patients, but little is known about its nature. Although many reports revealed that protein-bound uremic solutes induce reactive oxygen species production, the effects of these solutes on anti-oxidant level have not been well studied. Therefore, we examined the effects of protein-bound uremic solutes on glutathione levels. As a result, indoxyl sulfate, phenyl sulfate, and *p*-cresyl sulfate decreased glutathione levels in porcine renal tubular cells. Next we examined whether phenyl sulfate-treated cells becomes vulnerable to oxidative stress. In phenyl sulfate-treated cells, hydrogen peroxide induced higher rates of cell death than in control cells. Buthionine sulfoximine, which is known to decrease glutathione level, well mimicked the effect of phenyl sulfate. Finally, we evaluated a mixture of indoxyl sulfate, phenyl sulfate, and *p*-cresyl sulfate at concentrations comparable to the serum concentrations of hemodialysis patients, and we confirmed its decreasing effect on glutathione level. In conclusion, indoxyl sulfate, phenyl sulfate, and *p*-cresyl sulfate decrease glutathione levels, rendering the cells vulnerable to oxidative stress.

## Introduction

Oxidative stress is one of the hallmarks of chronic kidney disease (CKD) [1], and has attracted much attention as a mediator of cardiovascular disease (CVD) [2, 3], which is the leading cause of death in CKD patients on dialysis [4]. Oxidative stress can be driven by increases of pro-oxidants and/or decreases of anti-oxidants. Although the factors which induce an imbalance of the oxidant status in CKD patients are not fully known, uremic solutes have been suggested as being one of them [1].

In healthy individuals, uremic solutes are excreted in urine, whereas they accumulate in CKD patients [5]. Among over a hundred uremic solutes reported to date, protein-bound uremic solutes have attracted increasing attention in the last decade, because they are less efficiently removed by dialysis [6] and are suspected to contribute to CKD and CKD-related complications [7, 8].

Indoxyl sulfate (IS) is a representative protein-bound uremic solute [7]. Its concentration is reported to be elevated in CKD patients as disease progresses, and to be associated with CVD [9]. It is also reported that IS induces reactive oxygen species (ROS) production in various cell types [10, 11, 12], including renal tubular cells [13]. *Para*-cresyl sulfate (PCS) and indoleacetic acid (IAA) are also protein-bound uremic solutes, and have also been reported to induce ROS production in renal tubular cells [13, 14]. Although these studies demonstrate the influence of these protein-bound uremic solutes on oxidative stress in CKD patients through their induction of pro-oxidant production, very few studies addressing the impact of these solutes on anti-oxidant levels have been conducted.

Previously, we reported several protein-bound uremic solutes, including IS, as possible biomarkers for the effect of AST-120 [15], spherical activated carbon, which is a prescribed drug for CKD patients in Japan, Korea and Philippines. In that study, phenyl sulfate (PhS) was proposed as a novel biomarker for the effect of AST-120. We believe clarifying the impact of each uremic solute is paramount toward estimating the combined impact of these solutes in uremic state. Although a considerable amount of PhS has been detected in the serum of hemodialysis patients [16], it has been relatively less studied than IS, and therefore, its impact on biological systems is still unknown.

Here, we evaluated the effects of four protein-bound uremic solutes on intracellular glutathione, a major anti-oxidant molecule. We also examined whether pretreatment with these solutes would render cells vulnerable to oxidative stress.

## Materials and methods

### Cell culture

LLC-PK1, a porcine renal tubular epithelial cell line, was obtained from American Type Culture Collection (ATCC, Manassas, VA, USA). LLC-PK1 cells were maintained in Medium 199 (Mediatech, Manassas, VA, USA) supplemented with 10% FBS (Gibco, Life Technologies Japan, Tokyo, Japan).

### Reagents

Indoxyl sulfate was purchased from Biosynth (Staad, Switzerland). Phenyl sulfate and *p*-cresyl sulfate were synthesized at Eiweiss (Shizuoka, Japan). Indoleacetic acid was purchased from Tokyo chemical industry (Tokyo, Japan). Hydrogen peroxide and reduced glutathione were purchased from Wako Pure Chemical Industries (Osaka, Japan). Buthionine sulfoximine was purchased from Cayman Chemical (Ann Arbor, MI, USA). Meta-phosphoric acid was purchased from Sigma-Aldrich (St. Louis, MO, USA).

### Treatment of cells

Cells were seeded into appropriate culture dishes or culture plates at a density of 0.8 x 10^4^ cells/mL in Medium 199 supplemented with 10% FBS. After 24 hours, medium was replaced with Medium 199 supplemented with 2% FBS and indicated concentrations of protein-bound uremic solutes or buthionine sulfoximine. Cells were incubated for another 24 hours.

In some experiments, after a 24-hour incubation with protein-bound uremic solutes or buthionine sulfoximine, cells were further treated with hydrogen peroxide for 5 hours, in the absence or presence of reduced glutathione.

### Determination of the glutathione level

The total glutathione level was measured by using a Glutathione Assay Kit (Cayman Chemical).

After treatment of cells, 5 x 10^5^ cells were lysed in 50 μL of 5% meta-phosphoric acid. The total glutathione level in 50 μL of the lysate was expressed as molar concentration of reduced glutathione.

### Cellular viability

Cellular viability was determined by using the Cell Counting Kit-8 (Dojindo, Kumamoto, Japan), a water-soluble version of the methyl thiazolyl tetrazolium assay.

After treatment of cells, medium was replaced with medium containing the Cell Counting Kit-8 reagent. The cells were incubated for another 30 min and the optical density at 450 nm (OD450) was measured using a microplate reader (iMark^™^ Microplate Reader, Bio-Rad, Hercules, CA, USA). The OD450 of medium containing Cell Counting Kit-8 reagent without cells was measured, and subsequently subtracted from the OD450 value obtained from each sample. Cellular viability was expressed as percent of control.

### Apoptosis

After treatment of cells, culture medium that might contain non-adherent cells was harvested. Adherent cells were also harvested. These non-adherent cells and adherent cells were combined, and stained with FITC-conjugated annexin V and propidium iodide (TACS^™^ Annexin V-FITC Apoptosis Detection Kit, Trevigen, Gaithersburg, MD, USA). The stained cells were detected using a flow cytometer (FACS Calibur^™^, Becton, Dickinson and Company, Franklin Lakes, NJ, USA) and the data were analyzed using the FlowJo^™^ software (ver. 7.6.5, Tomy Digital Biology, Tokyo, Japan).

### Statistical analysis

Statistical analysis was performed by Student’s *t*-test, and p < 0.05 was considered as significant. All experiments were repeated at least twice, and representative data are shown.

## Results

### Effects of uremic solutes on glutathione levels

**Fig 1:**
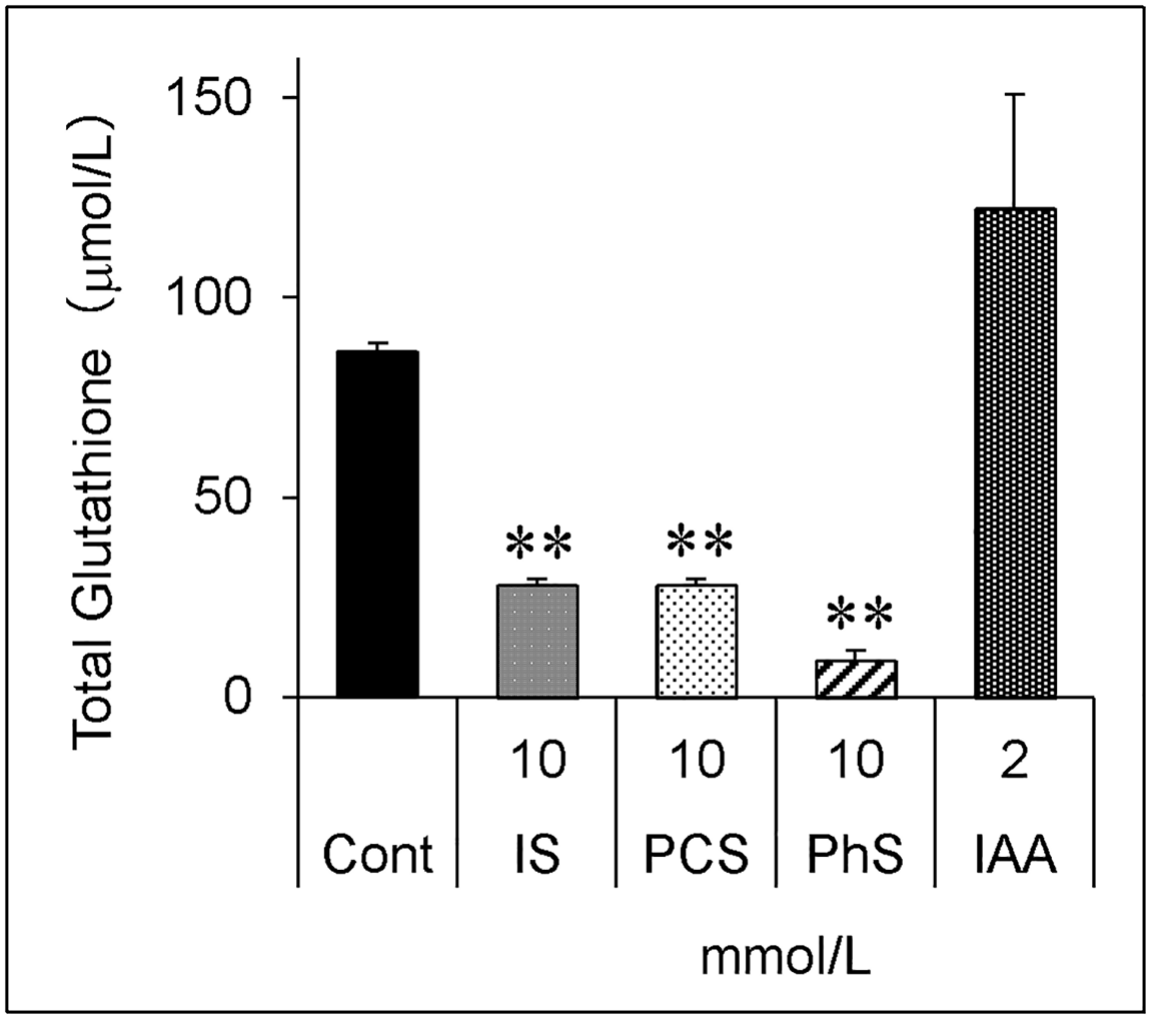
Effects of protein-bound uremic solutes on total glutathione level. Porcine renal tubular cells were treated with each of the four protein-bound uremic solutes for 24 hours. Cells were lysed and glutathione levels were measured using a Glutathione Assay Kit. The total glutathione level of 5 x 10^5^ cells in 50 μL lysate is expressed as molar concentration of reduced glutathione. Data are shown as mean ± S.D (n = 2). Data are representative of two independent experiments. Cont, control; IS, indoxyl sulfate; PCS, *p*-cresyl sulfate; PhS, phenyl sulfate; IAA, indoleacetic acid. Asterisks indicate significant difference compared to control (**p < 0.01).

Four protein-bound uremic solutes were tested for their effects on total glutathione levels (Fig 1). The concentration of each protein-bound uremic solute was set at a level high enough to inhibit cell growth or induce cell death, as determined in our previous study [17]. IS, PCS, and PhS significantly decreased glutathione levels, but IAA did not. PhS has the most prominent effect on the glutathione level, and therefore, was used for the following study.

**Fig 2:**
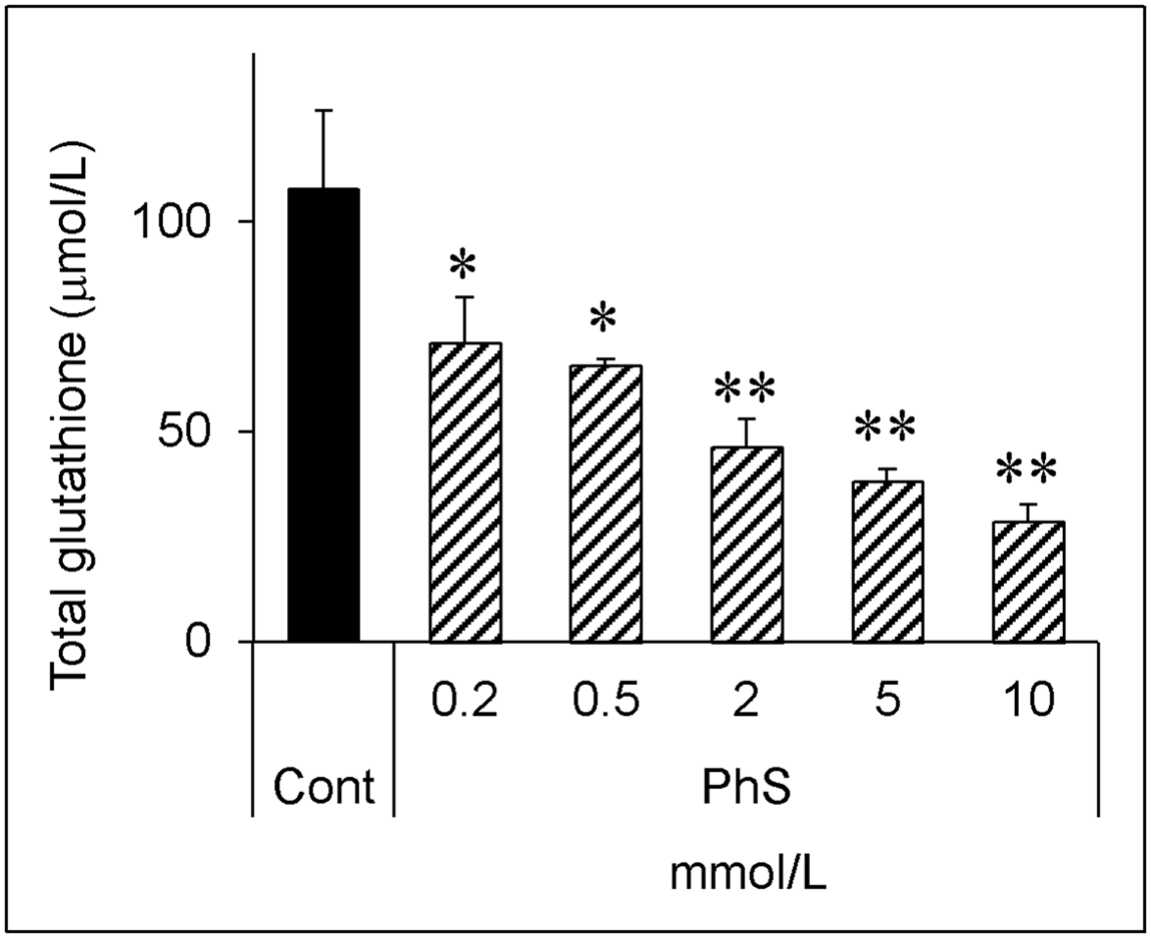
Dose-dependent effect of phenyl sulfate on total glutathione level. Porcine renal tubular cells were treated with indicated concentrations (0, 0.2, 0.5, 2, 5, 10 mmol/L) of phenyl sulfate for 24 hours. Cells were lysed and glutathione levels were measured as described in Fig 1. Data are shown as mean ± S.D (n = 3). Data are representative of two independent experiments. Cont, control; PhS, phenyl sulfate. Asterisks indicate significant difference compared to control (*p < 0.05, **p < 0.01).

PhS at 0.2 mmol/L significantly decreased the glutathione level, and higher concentrations decreased this level even further, in a dose-dependent manner (Fig 2). The measurement of oxidized glutathione showed a similar trend (unpublished observation).

### Impact of the decrease in the glutathione level

The decrease in the glutathione level is supposed to render cells vulnerable to oxidative stress. Thus, we evaluated the effect of hydrogen peroxide on cellular viability in the PhS-treated cells.

**Fig 3:**
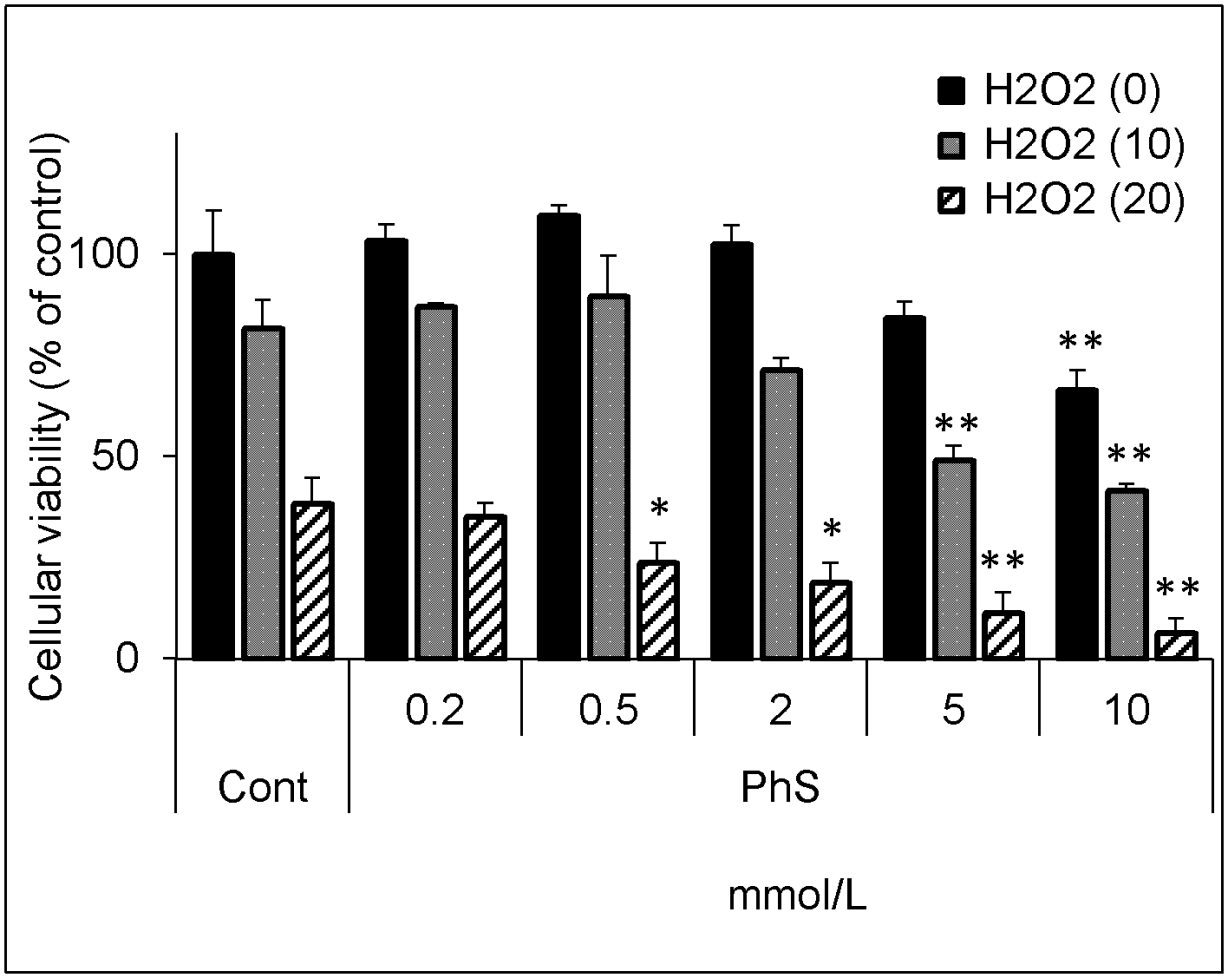
Effect of hydrogen peroxide on cellular viability in PhS-treated cells. Cells were treated with indicated concentrations (0, 0.2, 0.5, 2, 5, 10 mmol/L) of PhS for 24 hours and then treated with hydrogen peroxide (0, 10, 20 μmol/L) for 5 hours. After treatment, cellular viability was evaluated using Cell Counting Kit-8. Data are expressed as percent of control. Data are shown as mean ± S.D. (n = 3). Data are representative of two independent experiments. Cont, control; PhS, phenyl sulfate; H2O2, hydrogen peroxide. Asterisks indicate significant differences compared to corresponding controls (i.e. each black, grey, or hatched column was compared with its respective black, grey, or hatched control column) (*p < 0.05, **p < 0.01).

In control cells, 20 μmol/L of hydrogen peroxide decreased cellular viability by 62%, whereas in 0.5, 2, 5, or 10 mmol/L PhS-treated cells, the same concentration of hydrogen peroxide decreased cellular viability by 76%, 81%, 89%, and 94%, respectively (Fig 3). These differences between control cells and PhS-treated cells were statistically significant, although PhS itself, at 10 mmol/L, significantly decreased cellular viability by 34%. Furthermore, in control cells, 10 μmol/L of hydrogen peroxide decreased cellular viability by 18%, whereas in 5 or 10 mmol/L PhS-treated cells, the same concentration of hydrogen peroxide decreased cellular viability by 51 and 58%, respectively. These differences between control cells and PhS-treated cells were statistically significant.

**Fig 4:**
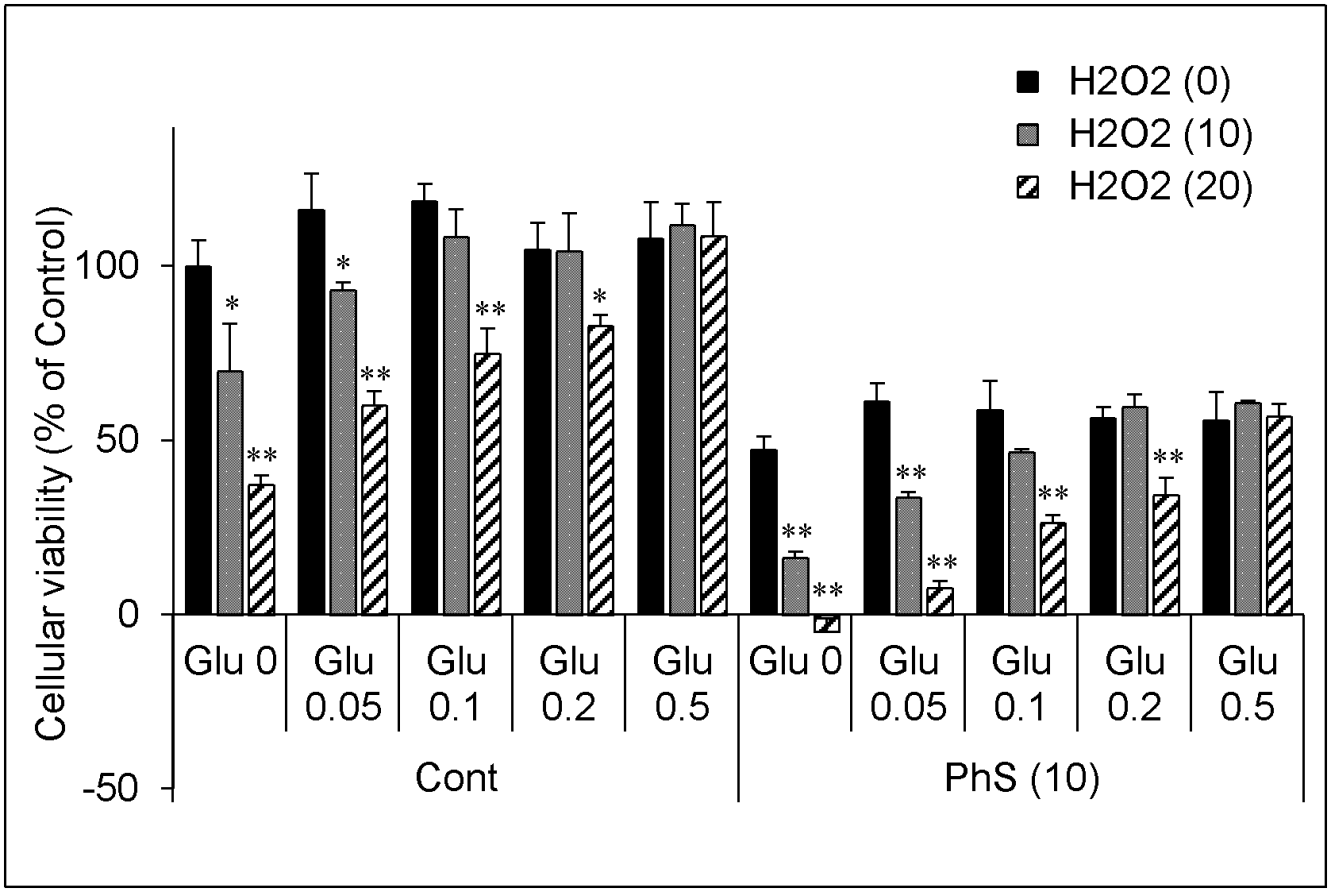
Effect of exogenous reduced glutathione on cellular vulnerability to hydrogen peroxide in PhS-treated cells. Cells were treated with 0 or 10 mmol/L of PhS for 24 hours and then treated with hydrogen peroxide (0, 10, 20 μmol/L) for 5 hours in the absence or presence of reduced glutathione (0, 0.05, 0.1, 0.2, 0.5 mmol/L). After treatment, cellular viability was evaluated as described in Fig 3. Data are expressed as percent of control. Data are shown as mean ± S.D. (n = 3). Data are representative of two independent experiments. Cont, control; PhS, phenyl sulfate; Glu, glutathione; H2O2, hydrogen peroxide. Asterisks indicate significant differences compared to respective 0 μmol/L of hydrogen peroxide (i.e. grey or hatched column was compared with respective black columns) (*p < 0.05, **p < 0.01).

These decreases in cellular viability were counteracted by the addition of exogenous reduced glutathione (Fig 4).

**Fig 5:**
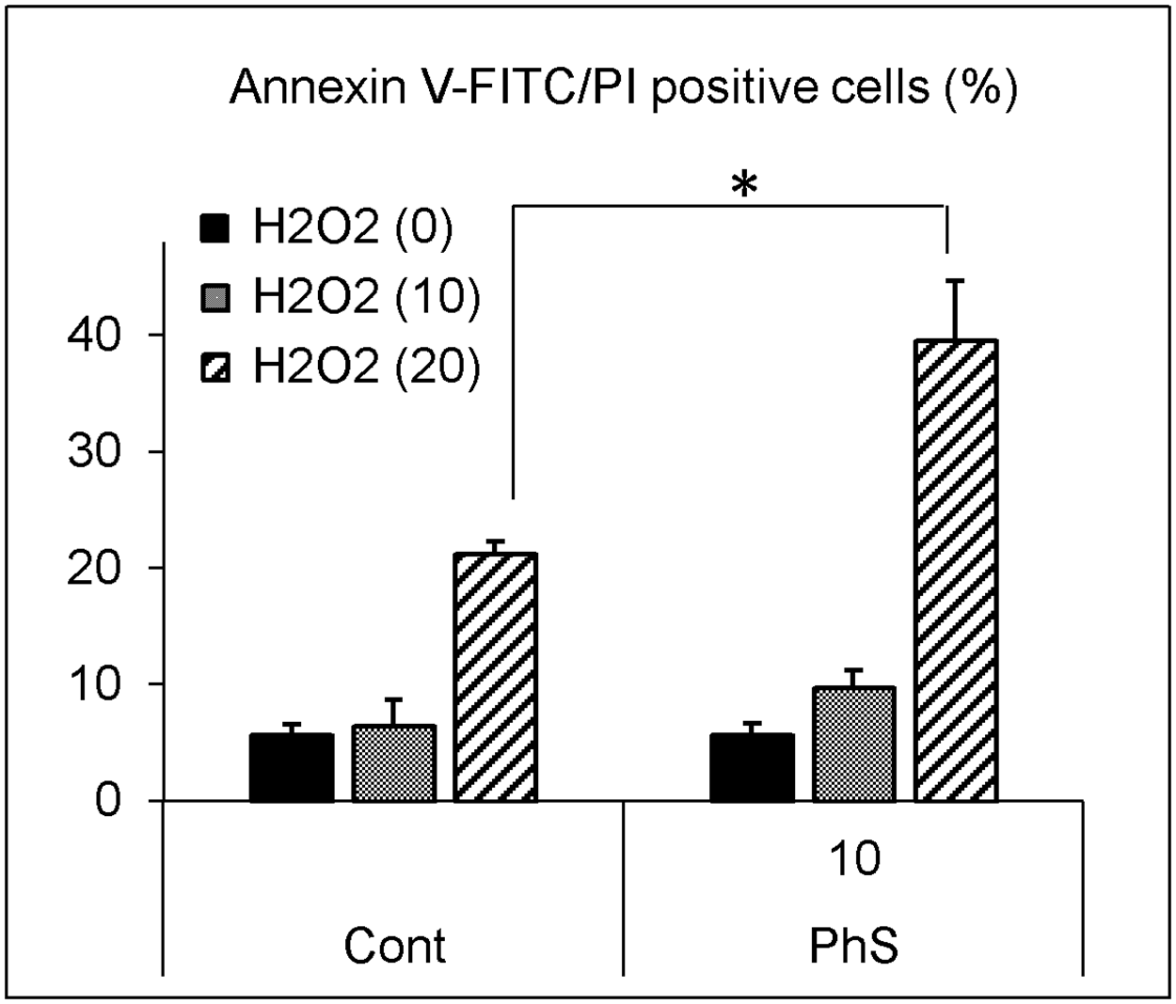
Effects of hydrogen peroxide on cell death in PhS-treated cells. Cells were treated with 0 or 10 mmol/L PhS for 24 hours and then treated with hydrogen peroxide (0, 10 or 20 μmol/L) for 5 hours. After treatment, cells were stained with annexin V-FITC and propidium iodide (PI). Percent of annexin V / PI-positive cells was analyzed and calculated. Data are shown as mean ± S.D. (n = 2). Data are representative of two independent experiments. Cont, control; PhS, phenyl sulfate; H2O2, hydrogen peroxide. Asterisk indicates significant difference between the indicated pair (*p < 0.05).

**Fig 6:**
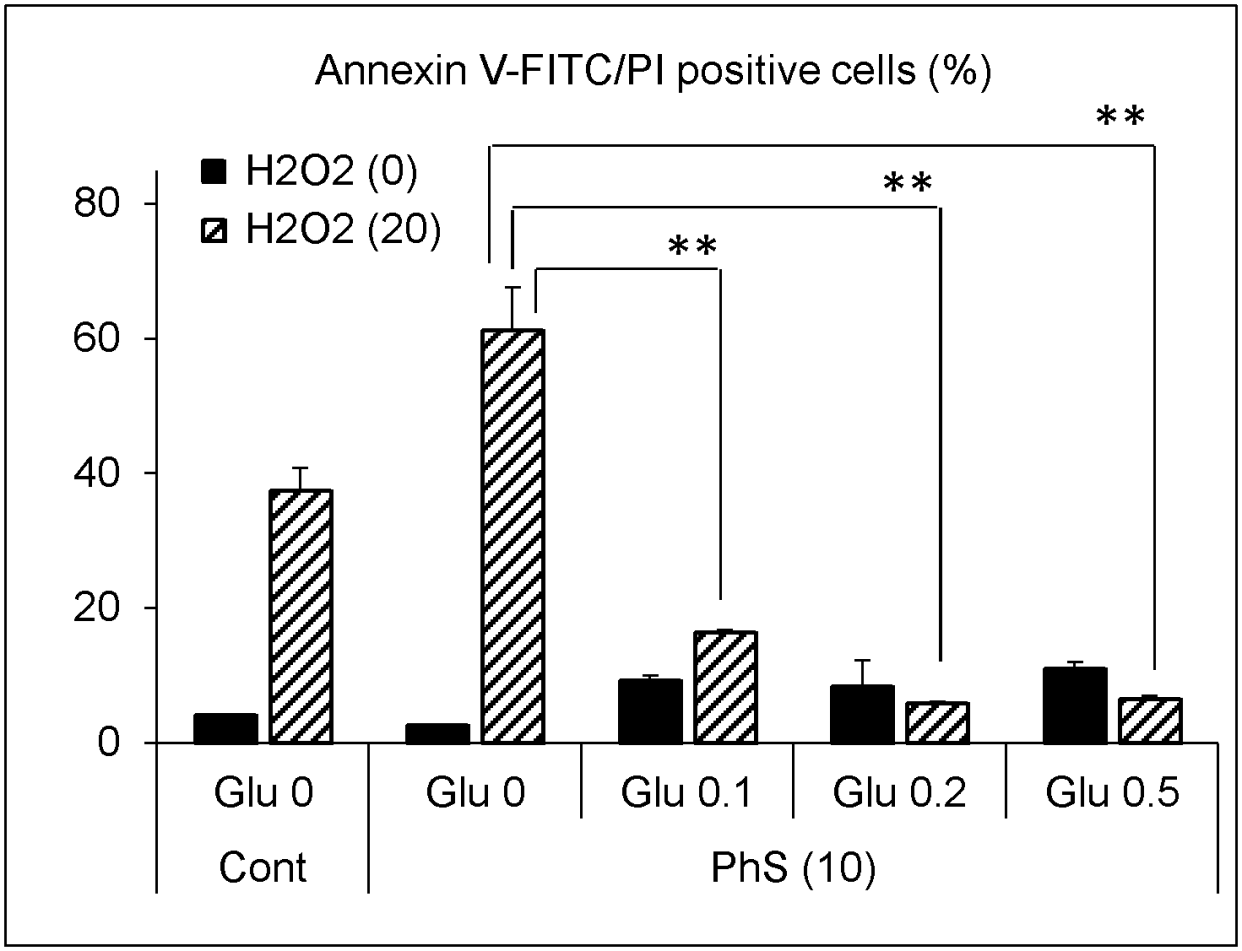
Effect of exogenous reduced glutathione on hydrogen peroxide-induced cell death in PhS-treated cells. Cells were treated with 10 mmol/L PhS for 24 hours and then treated with hydrogen peroxide (0 or 20 μmol/L) for 5 hours in the absence or presence of reduced glutathione (0, 0.1, 0.2, 0.5 mmol/L). After treatment, cell death was evaluated as described in Fig 5. Data are shown as mean ± S.D. (n = 2). Data are representative of two independent experiments. Cont, control; PhS, phenyl sulfate; Glu, glutathione; H2O2, hydrogen peroxide. Asterisks indicate significant differences between the indicated pairs (**p < 0.01).

Next, we examined the cell death rate in the same experimental setting. In control cells, 20 μmol/L of hydrogen peroxide increased Annexin V-FITC and propidium iodide double-positive cells to 21%, whereas in the 10 mmol/L PhS-treated cells, 40% of the cells stained positive, and this difference was statistically significant (Fig 5). The increase in cell death rate was counteracted by concomitant addition of exogenous reduced glutathione (Fig 6).

To confirm that the decrease in the glutathione level itself affects the cellular vulnerability to hydrogen peroxide, we used buthionine sulfoximine (BSO), which is an inhibitor of γ-glutamylcysteine synthetase [18], the rate-limiting enzyme for glutathione synthesis.

**Fig 7:**
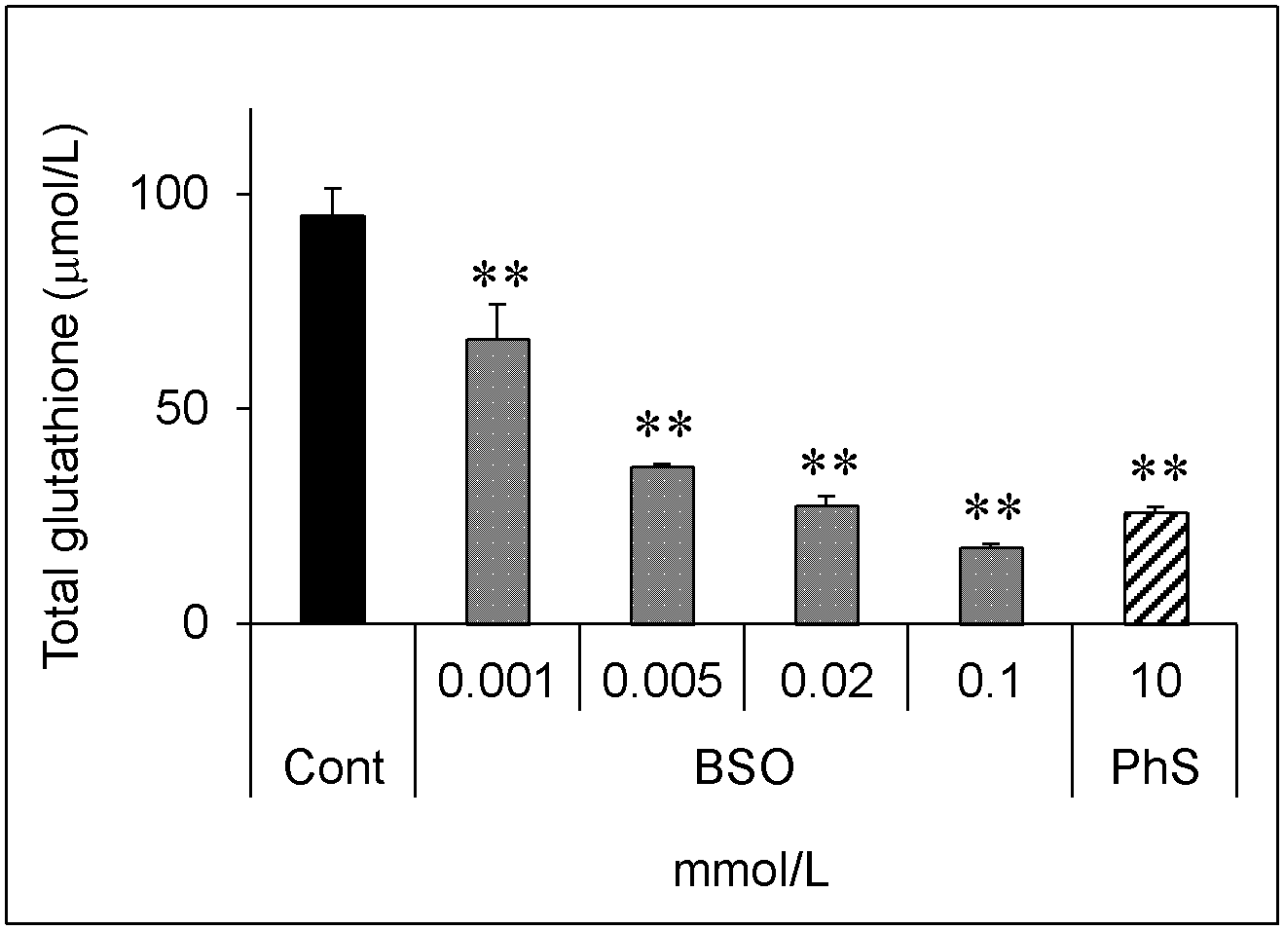
Dose-dependent effect of buthionine sulfoximine on total glutathione level. Cells were treated with indicated concentrations (0, 0.001, 0.005, 0.02, 0.1 mmol/L) of buthionine sulfoximine or 10 mmol/L PhS for 24 hours. Cells were lysed and glutathione levels were measured as described in Fig 1. Data are shown as mean ± S.D. (n = 3). Data are representative of two independent experiments. Cont, control; BSO, buthionine sulfoximine; PhS, phenyl sulfate. Asterisks indicate significant differences compared to control (**p < 0.01).

**Fig 8:**
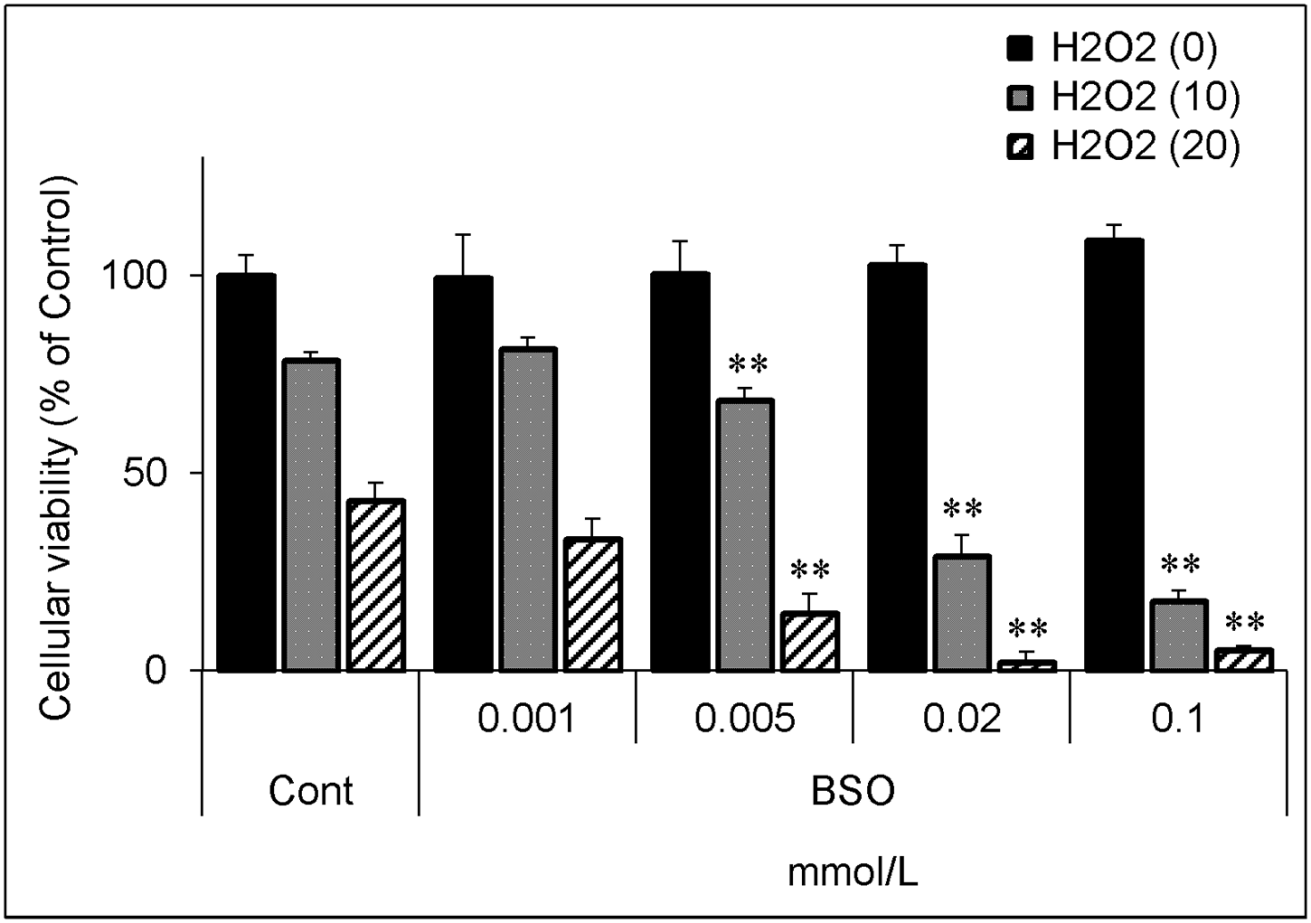
Effect of hydrogen peroxide on cellular viability in BSO-treated cells. Cells were treated with indicated concentrations (0, 0.001, 0.005, 0.02, 0.1 mmol/L) of buthionine sulfoximine for 24 hours and then treated with hydrogen peroxide (0, 10, 20 μmol/L) for 5 hours. After treatment, cellular viability was evaluated as described in Fig 3. Data are shown as mean ± S.D. (n = 3). Data are representative of two independent experiments. Cont, control; BSO, buthionine sulfoximine; H2O2, hydrogen peroxide. Asterisks indicate significant differences compared to corresponding controls (i.e. each black, grey, or hatched column was compared with its respective black, grey, or hatched control column) (**p < 0.01).

**Fig 9:**
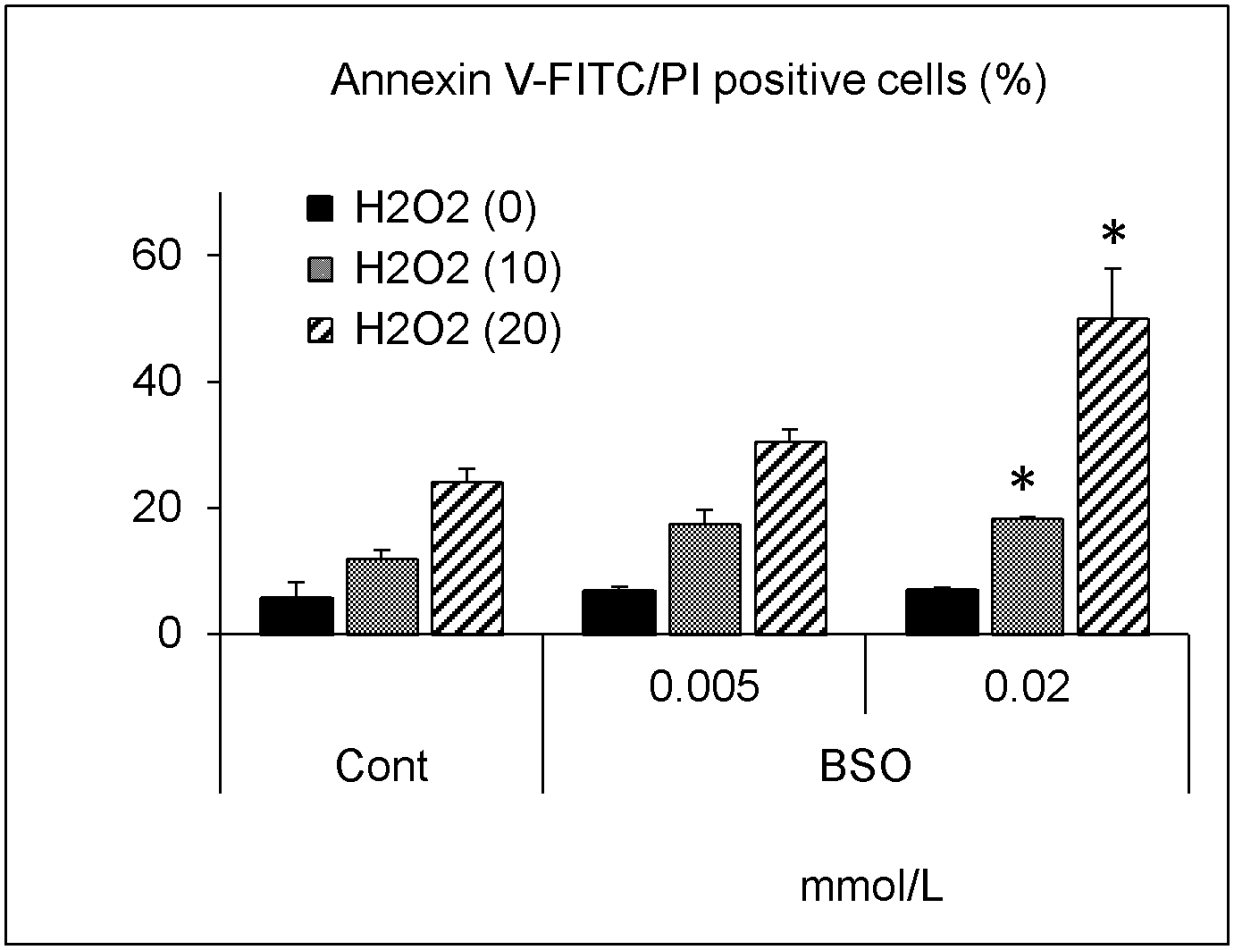
Effect of hydrogen peroxide on cell death in BSO-treated cells. Cells were treated with indicated concentrations (0, 0.005, 0.02 mmol/L) of buthionine sulfoximine for 24 hours and then treated with hydrogen peroxide (0, 10, 20 μmol/L) for 5 hours. After treatment, cell death was evaluated as described in Fig 5. Data are shown as mean ± S.D. (n = 2). Data are representative of two independent experiments. Cont, control; BSO, buthionine sulfoximine; H2O2, hydrogen peroxide. Asterisks indicate significant differences compared to corresponding controls (i.e. each black, grey, or hatched column was compared with its respective black, grey, or hatched control column) (*p < 0.05).

BSO significantly decreased the glutathione level in a dose-dependent manner (Fig 7). PhS at 10 mmol/L decreased the glutathione level to almost the same extent as BSO at 0.02 mmol/L. BSO itself, up to 0.1 mmol/L, did not affect cellular viability, but further decreased cellular viability upon hydrogen peroxide treatment (Fig 8). In a similar experiment, 0.02 mmol/L of BSO significantly augmented cell death induced by hydrogen peroxide (Fig 9).

### Effect of a mixture of uremic solutes on the glutathione level

Because the concentrations of the protein-bound uremic solutes tested in the aforementioned experiments were much higher than the serum concentrations reported in CKD patients [5, 16, 19], we next examined the effect of a mixture of protein-bound uremic solutes at pathological concentrations on the glutathione level.

**Fig 10:**
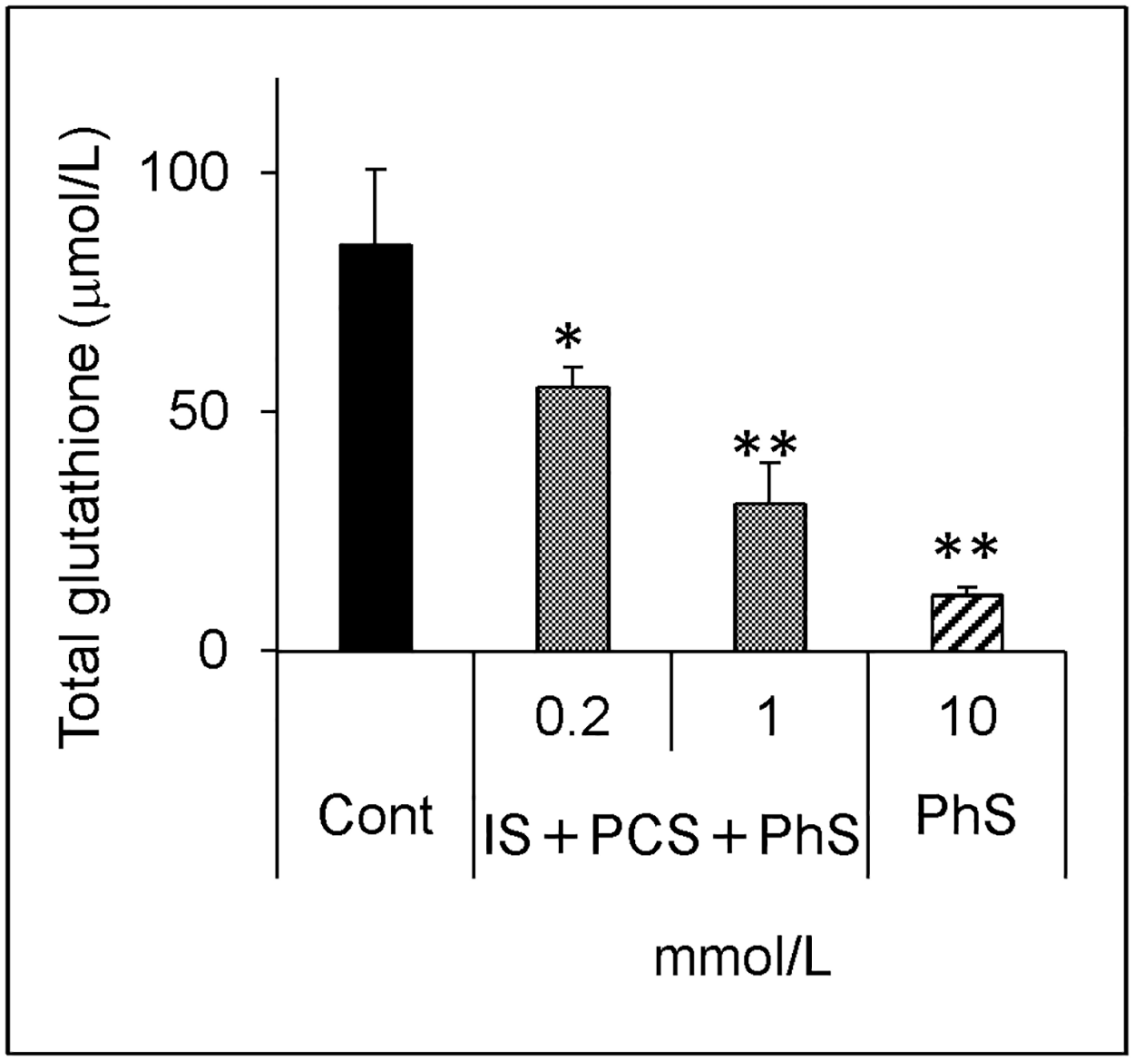
Effect of mixtures of three protein-bound uremic solutes on total glutathione levels. Cells were treated with medium containing indicated concentrations (0, 0.2, 1 mmol/L) of each protein-bound uremic solute or 10 mmol/L PhS for 24 hours. Cells were lysed and glutathione levels were measured as described in Fig 1. Data are shown as mean ± S.D. (n = 3). Data are representative of two independent experiments. Cont, control; IS, indoxyl sulfate; PCS, *p*-cresyl sulfate; PhS, phenyl sulfate. Asterisks indicate significant differences compared to control (*p < 0.05, **p < 0.01).

**Fig 11:**
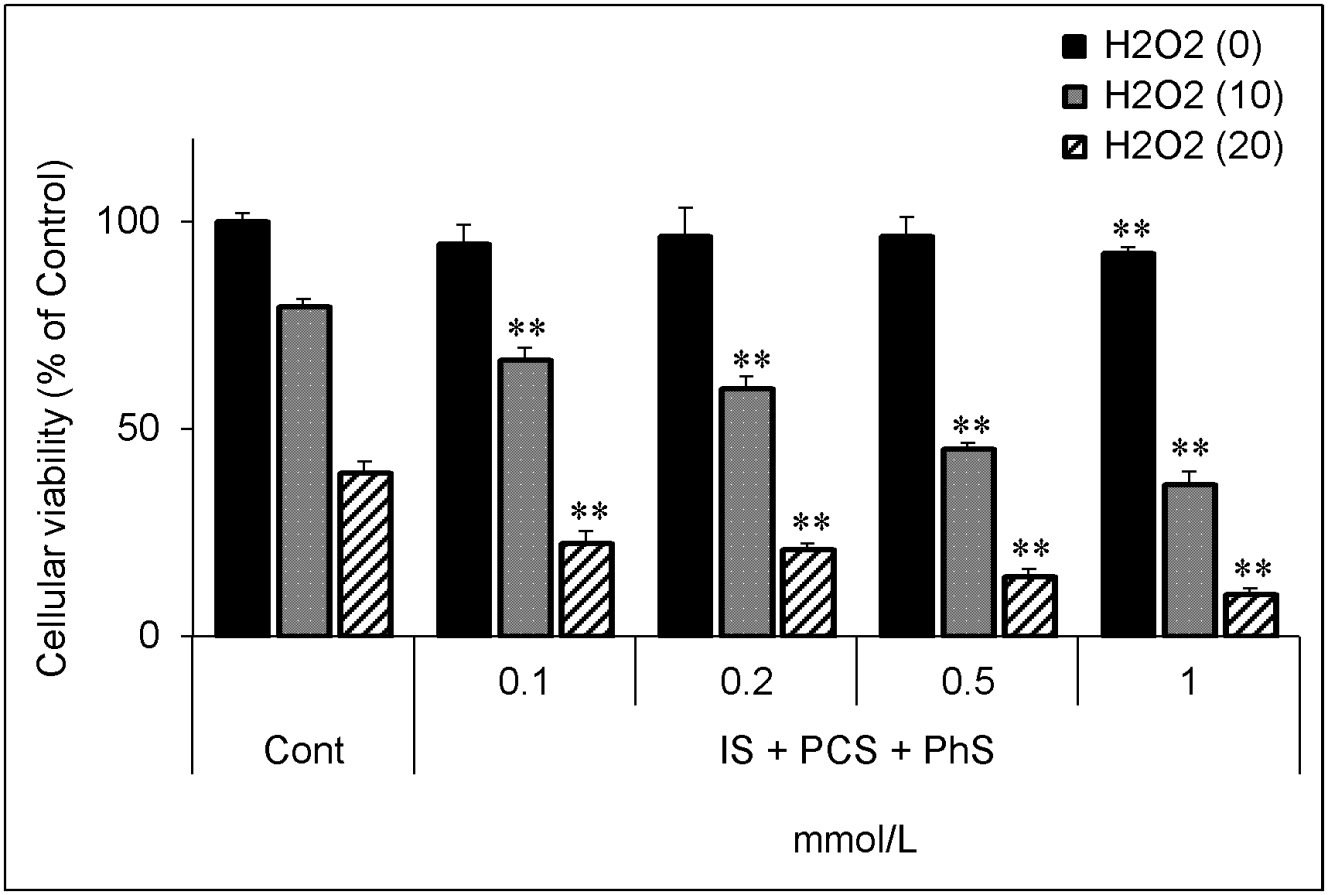
Effect of hydrogen peroxide on cellular viability in three uremic solutes-treated cells. Cells were treated with medium containing indicated concentrations (0, 0.1, 0.2, 0.5, 1 mmol/L) of each protein-bound uremic solute for 24 hours and then treated with hydrogen peroxide (0, 10, 20 μmol/L) for 5 hours. After treatment, cellular viability was evaluated as described in Fig 3. Data are shown as mean ± S.D. (n = 3). Data are representative of two independent experiments. Cont, control; IS, indoxyl sulfate; PCS, *p*-cresyl sulfate; PhS, phenyl sulfate; H2O2, hydrogen peroxide. Asterisks indicate significant differences compared to corresponding controls (i.e. each black, grey, or hatched column was compared with its respective black, grey, or hatched control column) (**p < 0.01).

The mixture, which was composed of IS, PCS, and PhS, each at 0.2 mmol/L, caused a significant decrease in the glutathione level, and a further decrease was observed on addition of 1 mmol/L of each solute (Fig 10). On addition of up to 0.5 mmol/L of each uremic solute, the mixture itself did not cause a significant decrease in cellular viability; however, pretreatment with the mixture resulted in a greater decrease in cellular viability upon hydrogen peroxide treatment (Fig 11).

## Discussion

To the best of our knowledge, this is the first study demonstrating the decreasing effects of protein-bound uremic solutes, especially PhS, on total glutathione levels, and their impacts on cellular vulnerability to oxidative stress in porcine renal tubular cells.

A previous report shows that IS decreases total glutathione levels in human umbilical vein endothelial cells [10]. The authors found that 0.5 or 1 mmol/L of IS can decrease the total glutathione level after 5 hours of incubation. Our results show that other protein-bound uremic solutes, such as PhS or PCS, can also induce such decrease.

In contrast to our results, or those obtained in the publication mentioned above, another study reports that 1 mmol/L of IS increases total glutathione levels in myocytes after a 24-hour incubation [20]. This increase was caused by the increased expression of γ-glutamylcysteine synthetase, a rate-limiting enzyme for glutathione production, as a result of anti-oxidative stress response to IS. Interestingly, we found that IAA decreases the total glutathione level after 5 hours of incubation (unpublished observation), but slightly increases it after 24 hours of incubation (Fig 1), compared with the corresponding control. This could also be a compensatory response; however, we did not investigate this phenomenon in detail here. In contrast to IAA, other protein-bound uremic solutes (IS, PhS and PCS) consistently decrease total glutathione levels after both 5 hours (unpublished observation) and 24 hours of incubation (Fig 1). Although the reasons for these discrepancies are unknown, the difference in cell types might be involved.

Oxidative stress has been reported in CKD patients [1] and is suspected to play a prominent role in disease progression and pathogenesis of its comorbidities, such as cardiovascular diseases [2, 3]. Oxidative stress is a pathological condition, which results from an imbalance between pro-oxidants and anti-oxidants, and several markers of oxidative stress in CKD have been proposed [21]. Glutathione is one of these markers, and is known as the most powerful anti-oxidant. It is reported that glutathione levels are reduced in erythrocytes or plasma of CKD patients [22, 23], especially in diabetic kidney disease patients [24–26], compared with healthy controls. Furthermore, lower glutathione levels have also been reported in kidney tissue of a CKD animal model [27–29]. Although contradictory results have been shown, i.e. constant or even higher levels of glutathione in CKD status have been reported [30–33], certain forces must exist, which contribute to the decrease in glutathione level. Our present study suggests protein-bound uremic solutes, IS, PhS, and PCS, as potential factors influencing these forces. Interestingly, AST-120, which has been confirmed to reduce the serum levels of these protein-bound uremic solutes in an animal model of CKD [15], is reported to increase the level of reduced glutathione and decrease that of oxidized glutathione in erythrocytes when administered to CKD patients [34].

Hydrogen peroxide is one of the reactive oxygen species, and is reported to be increased in plasma of patients on hemodialysis compared to healthy controls [35]. In animal models of CKD, increased levels of hydrogen peroxide in kidney or heart have also been reported [36, 37]. Furthermore, urea hydrogen peroxide, a stable form of hydrogen peroxide, has been shown to increase in kidney and heart of patients with CKD [38]. Although the exact concentration of hydrogen peroxide *in vivo* is still in debate, that found in plasma is expected to be 1~5 μmol/L at normal conditions, and may increase in case of pathological conditions [39].

Regarding protein-bound uremic solutes, the concentration that should be applied in *in vitro* studies was discussed in a literature review [40]. One of the suggested value to consider as appropriate for such studies is the highest individual value of serum concentration in uremic patients. In this review, the values were reported as 236 mg/L (1108 μmol/L) for IS and 105 mg/L (558.5 μmol/L) for PCS. Furthermore, in our previous study, we reported the highest individual values of serum concentrations in hemodialyzed patients as 268.5, 580.3 and 259.8 μmol/L for IS, PCS, and PhS, respectively [16]. Alternatively, the mean value of serum concentration in uremic patients is also thought to be an important measure. For IS and PCS, we and others reported mean values of serum concentrations of 90.1 ~ 140.4 μmol/L and 111.2 ~ 297.3 μmol/L, respectively [5, 16, 19]. For PhS, we reported a mean value of serum concentration of 77.6 μmol/L [16], which represents, to the best of our knowledge, the only value available in the literature. The concentrations tested in this study (Figs 10 and 11) are well comparable to those reported previously and are comprised within the accepted range.

In conclusion, a mixture of IS, PhS, and PCS at pathophysiological concentrations can decrease the total glutathione level and render cells vulnerable to oxidative stress. A future study will be necessary to confirm the *in vivo* relevance of these findings and clarify the mechanisms driving the decrease in glutathione levels under the effects of these protein-bound uremic solutes.
